# Involvement of NLRP3 inflammasome pathway in the protective mechanisms of ferulic acid and p-coumaric acid in LPS-induced sickness behavior and neuroinflammation in mice

**DOI:** 10.1007/s00210-023-02743-8

**Published:** 2023-09-27

**Authors:** Manas Kinra, Niraja Ranadive, Madhavan Nampoothiri, Devinder Arora, Jayesh Mudgal

**Affiliations:** 1 Department of Pharmacology, Manipal College of Pharmaceutical Sciences, Manipal Academy of Higher Education, Manipal, 576104 Karnataka India; 2 School of Pharmacy and Medical Sciences, Griffith University, Gold Coast Campus, Gold Coast, QLD 4222 Australia

**Keywords:** Ferulic acid, p-Coumaric acid, NLRP3 inflammasome, Sickness behavior, Neuroinflammation, Cytokines

## Abstract

Ferulic acid (FA) and p-coumaric acid (PCA) are abundantly present in commonly consumed food and beverages. Being polyphenolic compounds, they have been explored for their antioxidant and anti-inflammatory properties. Based on our previous study, we selected these two compounds to further investigate their potential in lipopolysaccharide (LPS)-induced sickness behavior and the ensuing neuroinflammation by specifically focusing on the NLRP3 inflammasome pathway. Male Swiss albino mice were divided into nine groups (n = 6) consisting of Normal Control, LPS, fluoxetine (FLX), FA40, FA160, FA640, PCA40, PCA160, and PCA640 respectively. Each group received respective FA or PCA treatment except Normal Control and LPS, which received the vehicle, carboxymethylcellulose 0.25% w/v. All groups were challenged with LPS 1.5 mg/kg, intraperitoneally except the Normal Control group, which received saline. Behavioral assessments were performed between 1–2 h, and the whole brains were collected at 3 h post-LPS administration. LPS-induced sickness behavior was characterized by significantly reduced spontaneous activity and high immobility time. The expression of NLRP3, ASC, caspase-1 and IL-1β was significantly increased, along with the levels of brain IL-1β suggesting the assembly and activation of NLRP3 inflammasome pathway. Furthermore, the major cytokines involved in sickness behavior, IL-6 and TNF-α were also significantly elevated with the accompanied lipid peroxidation. The results of this study emphasize that within the employed dose ranges of both FA and PCA, both the compounds were effective at blocking the activation of the NLRP3 inflammasome pathway and thereby reducing the release of IL-1β and the sickness behavior symptoms. There was a prominent effect on cytokine levels and lipid peroxidation as well.

**Graphical abstract:**

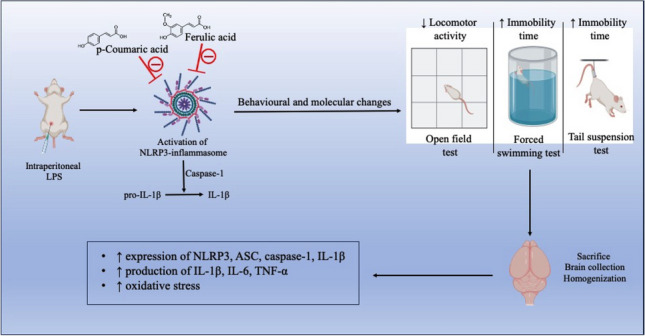

## Introduction

Neuroinflammation is a complex process that involves the activation of the innate immune system in response to various stimuli such as infection, injury, or neurodegenerative disorders (Amor et al. 2010). One important component of the innate immune system is the NLRP3 inflammasome, which plays a crucial role in the regulation of neuroinflammation (Lin and Mei 2020). The NLRP3 inflammasome is a protein complex that is composed of the NOD-like receptor family pyrin domain containing 3 (NLRP3) protein, the adaptor protein ASC, and the effector protein caspase-1. The activation of the NLRP3 inflammasome pathway is a critical step in the maturation and secretion of IL-1β and IL-18, which are involved in the progression of neuroinflammatory disorders (He et al. 2016). In the central nervous system (CNS), the NLRP3 inflammasome is activated in response to a variety of stimuli, including pathogen-associated molecular patterns (PAMPs), damage-associated molecular patterns (DAMPs), and environmental toxins (Jo et al. 2016). Excessive or prolonged activation of the NLRP3 inflammasome can lead to chronic neuroinflammation, which is associated with the pathogenesis of neurodegenerative diseases such as Alzheimer's disease (Feng et al. 2020), Parkinson's disease (Wang et al. 2019), and multiple sclerosis (Olcum et al. 2020). In experimental settings, the NLRP3 inflammasome pathway can be activated using several techniques, however, use of lipopolysaccharide (LPS) is one of the most potent and common methods to study the pathway (Xie et al. 2014). LPS is a component of the outer membrane of Gram-negative bacteria that is recognized by the immune system as a PAMP. LPS activates the innate immune response by binding to Toll-like receptor 4 (TLR4) on immune cells, leading to the activation of downstream signaling pathways and the production of pro-inflammatory cytokines. One important downstream signaling pathway activated by LPS is the NLRP3 inflammasome pathway (Yang et al. 2020). In mice, LPS administration has been shown to induce sickness behavior (Henry et al. 2008). Sickness behavior in mice is characterized as the presence of symptoms such as decreased locomotor activity, anorexia, anhedonia, hyperalgesia, etc. and is believed to be an adaptive response to neuroinflammation (Biesmans et al. 2013). Further, recent evidence suggests that activation of the NLRP3-inflammasome pathway and subsequent production of cytokines can weaken and disrupt the integrity of the blood brain barrier (BBB). Studies have shown that NLRP3 activation can disrupt the integrity of the BBB by inducing the production of matrix metalloproteinases (MMPs), ROS, and oxidative stress, which can lead to the breakdown of tight junctions and the loss of BBB integrity (Chen et al. 2021).

FA is a well-known oxidative radical scavenger present in commonly consumed fruits, beverages, and vegetables (Mancuso and Santangelo 2014; Contardi et al. 2021; Stompor-Gorący and Machaczka 2021). Like other polyphenolic compounds, the antioxidant properties of FA have been extensively reported (Graf 1992; Zduńska et al. 2018). Moreover, FA has been observed to be effective in depression (Singh et al. 2017; Liu et al. 2017; Zeni et al. 2017; Zheng et al. 2019), neuroinflammation (Sun et al. 2021; Jiang et al. 2021; Singh 2017; Rehman et al. 2019), neurodegeneration (Chaudhary et al. 2019; Singh et al. 2017; Sultana 2012), and cancer (Zduńska et al. 2018). FA inhibits NF-κB, iNOS, COX-2 and modulates LPS-induced TLR4 pathway (Rehman et al. 2019; Jung et al. 2009).

PCA is a phenolic acid and can be subsequently transformed into other bioactive molecules such as phenolic acids (e.g. caffeic acid, ferulic acid, chlorogenic acid and sinapic acid), flavonoids, lignin precursors and other secondary metabolites. PCA has been shown to express various biological potentials including antioxidant, anti-inflammatory, antimutagenic, anti-ulcer, antiplatelet and anti-cancer properties. In addition, it has been reported to mitigate atherosclerosis, oxidative cardiac damage, UV-induced damage to ocular tissues, neuronal injury, anxiety, gout, and diabetes-induced neurodegeneration (Abdel-Moneim et al. 2017). Furthermore, PCA downregulates NF-κB and MAPK pathways, inhibits iNOS, COX-2, TNF-α, IL-1β and TNF-α expression at mRNA as well as protein levels (Kheiry et al. 2019; Pragasam et al. 2013; Rafiee et al. 2020).

With the *in-silico* and *in-vitro* assessment of various phenylpropanoic acid derivatives including, caffeic acid, caffeic acid phenethyl ester, chlorogenic acid, ethyl-p-methoxycinnamate, p-methoxy cinnamic acid, cinnamic acid, FA and PCA, both FA and PCA were selected for *in-vivo* screening based on the supporting evidence from our previous study (Kinra et al. 2021). In our current study, we focused mainly on comparing the dose-dependent effects of both FA and PCA against the LPS-induced NLRP3-inflammasome pathway-mediated sickness behavior.

## Materials and methods

### Animals

Male Swiss albino mice, 25–30 g, were selected for the study. All experimental protocols were approved by Institutional Animal Ethics Committee of Kasturba Medical College, Manipal, MAHE (Approval number IAEC/KMC/113/2020 dated 7^th^ November 2020). Animals were housed in controlled conditions of temperature (23 ± 2 °C) and humidity (50 ± 5%) with 12-h light & dark cycle. Animals were provided with food and water ad libitum. All experiments were carried out as per the Committee for the Purpose of Control and Supervision of Experiments on Animals (CPCSEA) guidelines.

### Chemicals and reagents

All the chemicals used in this study were analytical grade. FA, PCA, LPS (*Escherichia coli* 0111:B4), and 2-thiobarbituric acid (TBA) were purchased from Sigma-Aldrich Co. LLC (St Louis, MO, USA). Carboxymethylcellulose (CMC), sodium dihydrogen phosphate anhydrous, disodium hydrogen phosphate anhydrous and trichloroacetic acid were purchased from Merck Millipore Corporation (Merck KGaA, Darmstadt, Germany). ECL reagent, BCA protein estimation kit, ELISA kits, acrylamide, antibodies (NLRP3: Cat. No. PA579740; ASC: Cat. No. PA588132; caspase-1: Cat. No. PA587536; IL-1β: Cat. No. PA5105048, α-tubulin: Cat. No. PA585978; secondary antibody DOXRB Alex fluor plus 488: Cat. No. A32790), PVDF membrane, stacking buffer, and running buffer were purchased from ThermoFisher Scientific (Waltham, MA, USA). Blocking-grade blocker (nonfat dry milk) was obtained from Bio-Rad Laboratories (CA, USA).

### Treatments and grouping

Animals were randomly assigned into nine groups (n = 6 each), including group I: Normal Control; group II: LPS; group III, IV and V: Ferulic acid (FA40, FA160 and FA640 respectively); group VI, VII and VIII: p-Coumaric acid (PCA40, PCA160 and PCA640 respectively); group IX: fluoxetine (FLX). All the treatments were administered by oral route (*p.o.*), whereas LPS was administered by intraperitoneal (*i.p.*) injection. Normal Control and LPS groups received vehicle, carboxymethylcellulose (CMC, 0.25% w/v) at the dose of 10 ml/kg*.* FA40, FA160 and FA640 groups were treated with FA at the dose of 40, 160 and 640 mg/kg*.* PCA40, PCA160 and PCA640 groups were treated with PCA at the dose of 40, 160 and 640 mg/kg*.* FLX was administered at a dose of 20 mg/kg*.* All the treatments were administered once in the morning. All the groups except Normal Control, were injected with 1.5 mg/kg LPS*.* Behavioral assays were performed within 1–2 h of LPS administration and were video recorded. At the end of the behavioral estimations, animals were sacrificed by overdose of anesthesia (thiopentone sodium 100 mg/kg) and the whole brain was isolated carefully. The samples were stored at -80 °C until further estimations.

### Behavioral assays

Open field test (OFT) was used to assess the spontaneous locomotor activity (LMA) and the exploratory behavior. Whereas the forced swim test (FST), and tail suspension test (TST) were employed for behavioral despair induced by LPS administration. The assays were performed by following the standard procedures as described in detail earlier (Basu Mallik et al. 2016; Mudgal et al. 2019, 2020). Briefly, LMA was assessed in a clean glass open field arena (30 × 30 × 60 cm) with the number of square crossings into 9 virtual quadrants (10 × 10 cm each). FST was assessed by the total immobility time over the 5 min of observational period in a transparent plexiglass cylinder (30 × 20 cm), whereas TST was calculated as the immobility time when the animals were individually hung for a period of 5 min at 15 cm away from the nearest surface.

### Estimation of changes in cytokine levels in brain

The proinflammatory cytokines including, TNF-α, IL-6 and IL-1 β were estimated using commercially available enzyme-linked immunosorbent assay (ELISA) kits (Invitrogen, ThermoFisher Scientific, USA) as per the manufacturer’s instructions.

### Estimation of changes in expressed protein levels in brain

Mouse brain was homogenized in RIPA buffer with a protease-phosphatase inhibitor cocktail. The homogenate was sonicated and then centrifuged at 7000 xg for 20 min at 4 °C and supernatant was collected. The supernatant was used to perform total protein estimation by BCA method using the commercial kit (Invitrogen, ThermoFisher Scientific, USA). The samples were loaded into acrylamide gel (stacking gel: 4% w/v; resolving gel: 8% for NLRP3 and 14% for all other proteins) at a uniform loading concentration of 15 μg. The gel was run for 90 min at 100 V of uniform voltage. Semi-dry transfer was performed on PVDF membrane. The membrane was blocked using a blocker for 1 h. Post blocking, the membrane was washed thoroughly and then incubated with primary antibody overnight. All primary antibodies were used at a dilution of 1:500. After incubation with primary antibody, membrane was washed, incubated with secondary antibody, at a dilution of 1:10,000 for 1 h and then washed again. The membrane was then developed using ECL reagent and chemiluminescence was captured using a chemiluminometer. The protein bands were normalized against the individual α-tubulin and relative expression was calculated.

### Estimation of oxidative stress in mouse brain

Lipid peroxidation was quantified by measuring the malondialdehyde (MDA) levels in brain homogenates as described earlier (Janero 1990; Khan et al. 2013). The levels of MDA formed after complexation of oxidised lipids with TBA were measured spectrophotometrically at 532 nm and expressed as nmoles/mg of protein. Total protein estimation was carried out using commercial kit (Invitrogen, ThermoFisher Scientific, USA), as per the manufacturer’s instructions.

### Statistical analysis

Data were expressed as Mean ± SEM and analyzed using GraphPad Prism v9.3.0 (GraphPad Prism software, San Diego, CA, USA). All the parameters were analyzed by one-way analysis of variance (ANOVA) followed by Dunnett’s multiple comparison test. “p” value of < 0.05 was considered to be statistically significant.

## Results

### Effect of FA and PCA on LPS-induced sickness behavior

**Fig. 1:**
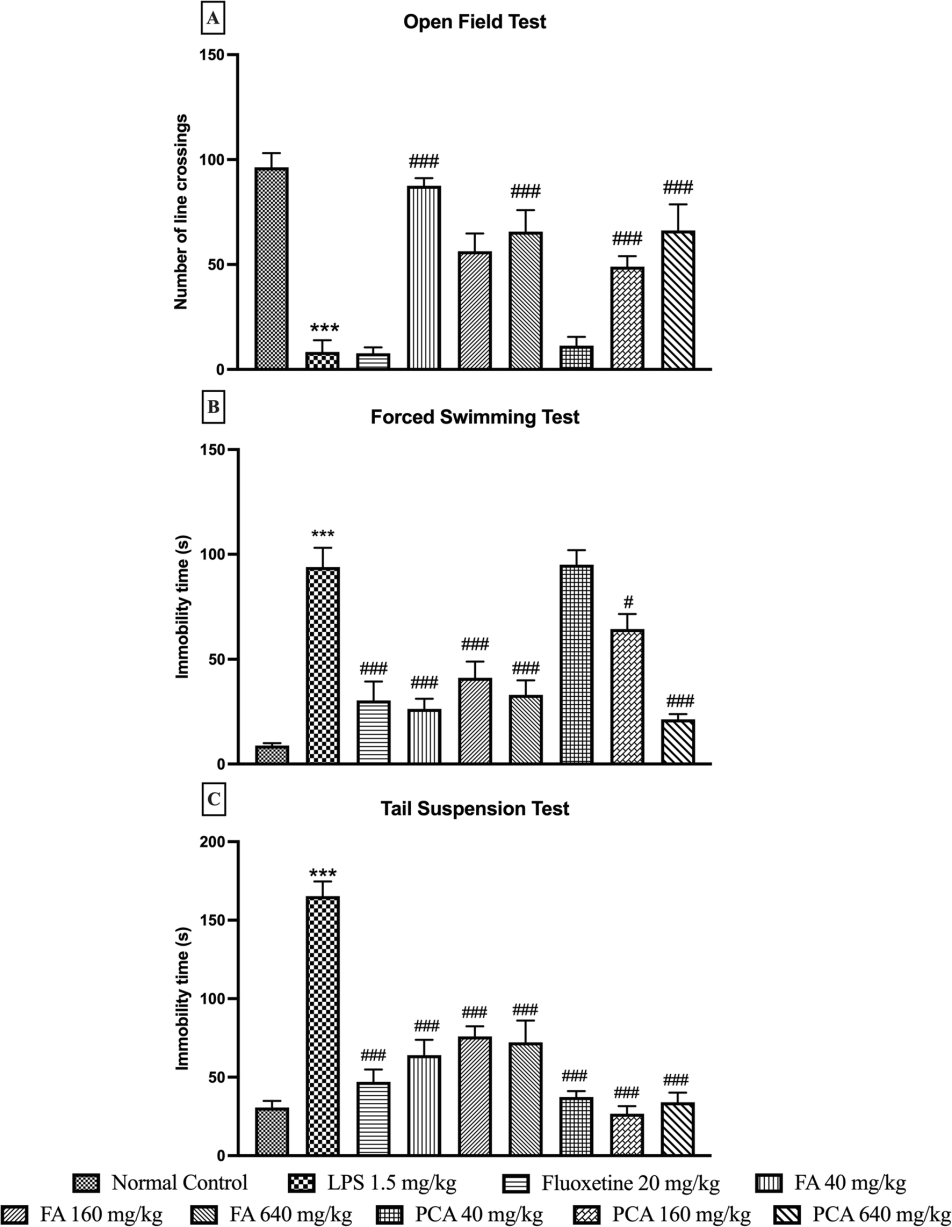
Effect of ferulic acid (FA40, 160, 640 mg/kg) and p-Coumaric acid (PCA40, 160, 640 mg/kg) on LPS-induced changes in (**A**) number of line crossings (n = 4–6); immobility time (s) as observed in (**B**) FST (n = 6); and (**C**) TST (n = 6). *** represents significant at p < 0.001 compared to Normal Control; #, ### represents significant at p < 0.05, p < 0.001 compared to LPS. The data is represented as Mean ± SEM

The spontaneous activity was significantly reduced by LPS administration as observed by the decreased number of line crossings (8.33 ± 5.61 vs 96.33 ± 6.80 of Normal Control group). Pretreatment of the animals with all three doses of FA, PCA160 and PCA640 significantly improved this LPS-induced reduction in LMA (p < 0.0001, F [8, 43] = 25.51, Fig. 1A). PCA40 (11.33 ± 4.18) and fluoxetine (8.33 ± 6.8) did not show any significant change in the LMA as compared to LPS treated group.

Furthermore, LPS treated animals expressed a significantly increased immobile state as observed in the FST immobility time (93.83 ± 9.16 s LPS vs 8.83 ± 1.14 s of Normal Control group). All the three doses of FA, positive control of FLX and the PCA160 and PCA640 showed a significant improvement in the immobility time as observed in the forced swim assay (p < 0.0001, F [8, 45] = 21.75 Fig. 1B). PCA40 did not show any significant effect on LPS-indued immobility time.

The immobility time was also significantly increased by the LPS administration in tail suspension assay (165.30 ± 9.40 s LPS vs 30.67 ± 4.23 s of Normal Control group). All the three doses of FA, PCA and FLX were very effective in reducing this impact on the immobility (p < 0.0001, F [8, 45] = 29.00, Fig. 1C).

### Effect of FA and PCA on LPS-induced changes in cytokines and MDA levels

The proinflammatory cytokines including, TNF-α, IL-6 and IL-1β were quantified in the brain homogenates (pg/mg protein). TNF-α (153.50 ± 10.61 vs 81.33 ± 2.19 of Normal Control group); IL-6 (130.20 ± 9.98 vs 47.17 ± 2.83 of Normal Control group) and IL-1β (111.70 ± 17.07 vs 8.77 ± 0.44 of Normal Control group) were significantly increased by LPS administration.

**Fig. 2:**
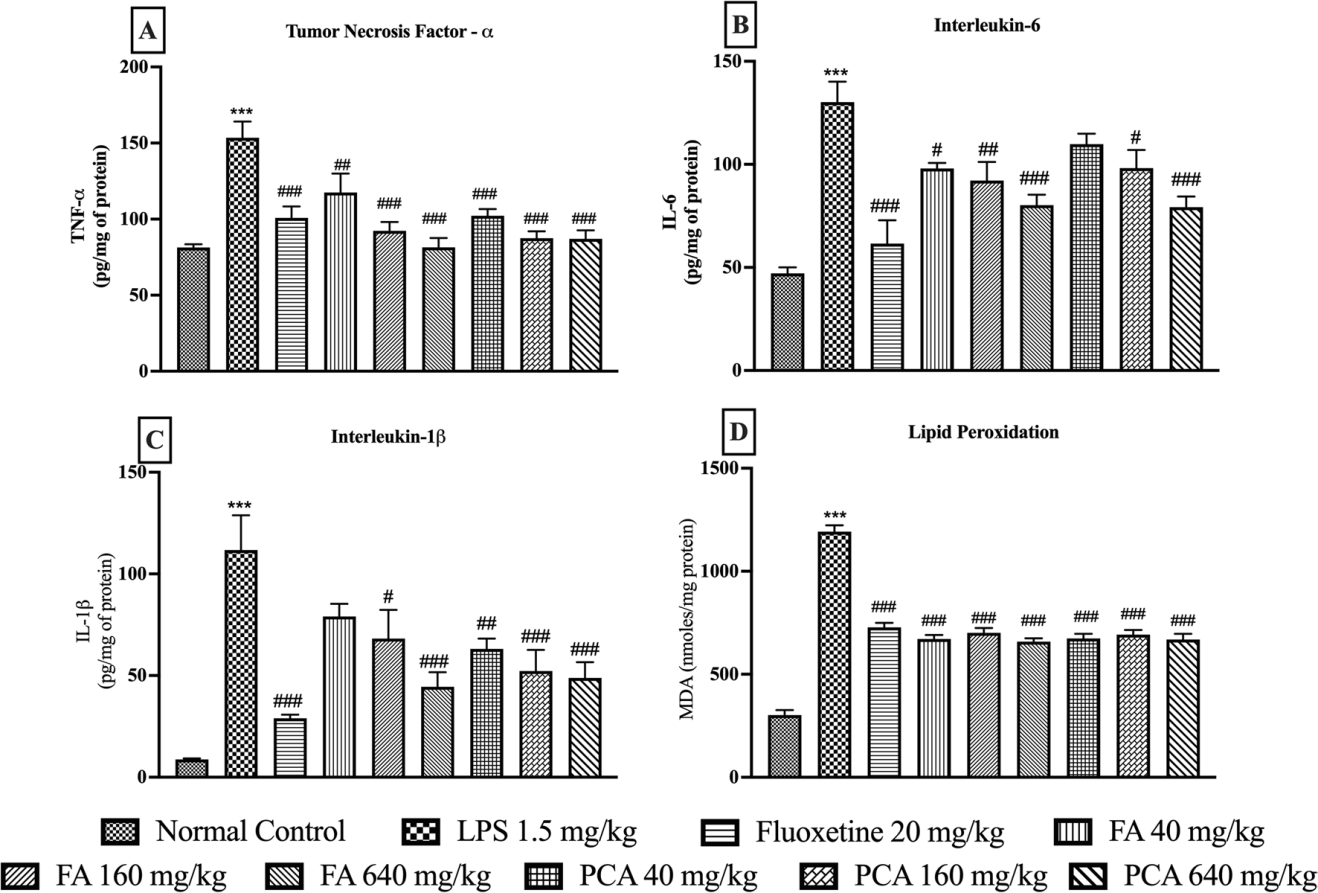
Effect of ferulic acid (FA40, 160, 640 mg/kg) and p-Coumaric acid (PCA40, 160, 640 mg/kg) on LPS-induced changes in brain homogenates; (**A**) TNF-α (pg/mg of protein) (n = 6); (**B**) IL-6 (pg/mg of protein) (n = 5–6); (**C**) IL-1β (pg/mg of protein) (n = 6); and (**D**) MDA (nmoles/mg protein) (n = 6). *** represents significant at p < 0.001 compared to Normal Control; #, ##, ### represents significant at p < 0.05, p < 0.01, p < 0.001 compared to LPS. The data is represented as Mean ± SEM

Pretreatment of the animals with all the doses of FA, PCA and the positive control FLX significantly reduced the LPS-induced increase in TNF-α (p < 0.001, F [8, 45] = 10.17, Fig. 2A). A similar trend was observed in both IL-6 (p < 0.0001, F [8, 42] = 11.91, Fig. 2B) and IL-1β (p < 0.0001, F [8, 45] = 10.07, Fig. 2C) levels, where both FA and PCA reduced the proinflammatory cytokines in a dose-dependent manner and these effects were comparable to the FLX treatment group. LPS administration significantly increased the lipid peroxidation in all the groups, as observed by an elevated MDA levels (nmoles/mg protein) (1193.00 ± 30.37 vs 301.80 ± 24.24 of Normal Control group). Pretreatment with both FA and PCA at all three doses, as well as FLX significantly reduced the MDA levels. (p < 0.0001, F [8, 45] = 93.30, Fig. 2D).

### Effect of FA and PCA on LPS-induced changes in protein levels of NLRP3-inflammasome pathway

**Fig. 3:**
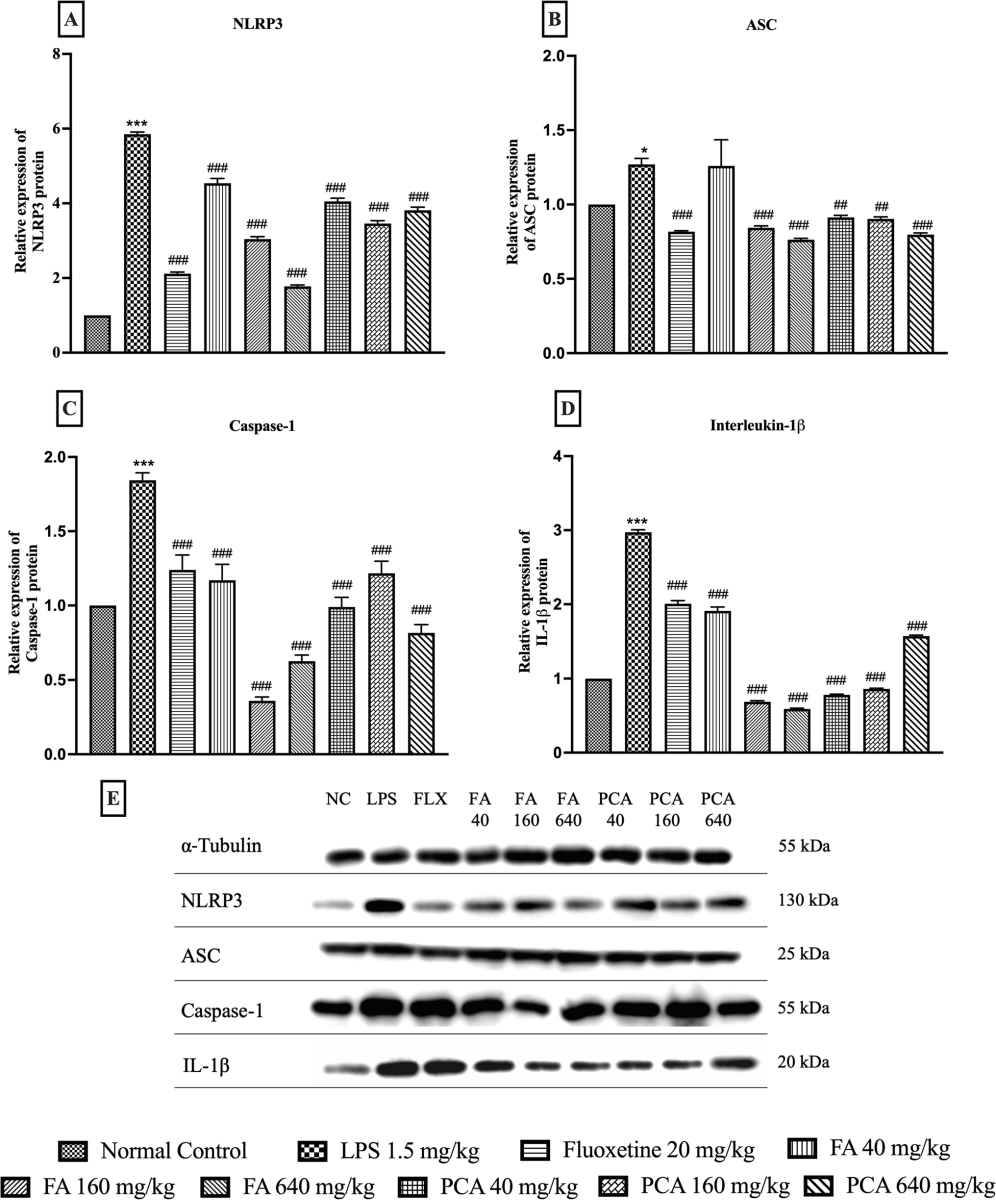
Effect of ferulic acid (FA40, 160, 640 mg/kg) and p-Coumaric acid (PCA40, 160, 640 mg/kg) on LPS-induced changes in protein expression of (**A**) NLRP3 (n = 3); (**B**) ASC (n = 3); (**C**) Caspase-1 (n = 3); (**D**) IL-1β (n = 3) and (**E**) representative blots. *, *** represents significant at p < 0.05, p < 0.001 compared to Normal Control; #, ##, ### represents significant at p < 0.05, p < 0.01, p < 0.001 compared to LPS. The data is represented as Mean ± SEM

LPS administration significantly increased the expression of NLRP3 (5.85 ± 0.06), ASC (1.27 ± 0.04), Caspase-1 (1.84 ± 0.04) and IL-1β (2.97 ± 0.03). The expression of NLRP3 was dose-dependently reduced by FA (p < 0.0001, F [8, 18] = 424, Fig. 3A), with FA640 having the maximum effect. Whereas the effect of PCA was comparable at all three doses. Expression of ASC was reduced by all the doses of PCA (p < 0.0001, F [8, 18] = 9.89, Fig. 3B), whereas the low dose, FA40 was ineffective in reducing the LPS-induced increase. Interestingly, all the doses of FA and PCA reduced Caspase-1 expression; (p < 0.0001, F [8, 18] = 39.49, Fig. 3C), however, irrespective of the dose-dependency at the lower doses and both FA and PCA640 were most effective. Finally, IL-1β expression (p < 0.0001, F [8, 19] = 948, Fig. 3D) was also significantly reduced with FA160, 640 and PCA40, 160 being the most effective doses.

## Discussion

Systemic administration of low doses of LPS causes an increase in peripheral as well as central cytokines which is bi-phasic and time-dependent. This “sickness behavior” lasts for 2–6 h and is characterized by an increase in cytokines, especially IL-1β, IL-6, and TNF-α in the peripheral circulation and subsequently in the brain. The animals administered with LPS also show a change in behavior which includes anhedonia, akinesia, decreased locomotor activity, decreased grooming, hyperalgesia, sleep disturbances, hunched posture, and anorexia (Zhao et al. 2019). After the initial phase of sickness behavior, the animals exhibit a “depressive-like state”, 24 h post-LPS administration (Dantzer et al. 2008). In this phase, the behavioral changes, especially locomotor activity starts reverting to normal but, the cytokine levels in the brain are still elevated.

These behavioral changes in mice can be quantified by using behavioral tests such as OFT, FST, and TST. OFT uses locomotor activity as a parameter, whereas FST and TST use the immobility state shown by animals during the task as predictors of sickness behavior. Since the behavioral changes in sickness behavior are accompanied by underlying neuroinflammatory state, the changes in levels of pro-inflammatory cytokines are indicative of the biochemical changes after LPS injection (Chan et al. 2020, Custódi et al. 2013).

LPS is a bacterial endotoxin from gram-negative bacteria that is recognized as a PAMP by the innate immune system, which activates the TLR4 receptors on the surface of the macrophages and initiates a cascade of signaling pathways (Lu et al. 2008). This cascade includes TRIF signaling which cleaves IκB to release active NF-κB (Han et al. 2004). Translocation of NF-kB into the nucleus then upregulates the production and release of pro-inflammatory cytokines. We have shown that LPS significantly increases the expression of NF-κB (Kinra et al. 2021). Simultaneously, TRIF signaling via MyD88 pathway (Sakai et al. 2017) activates the NLRP3-inflammasome pathway as well. Apart from activation of NLRP3 and ASC by simultaneous deubiquitination and ubiquitination respectively, the expression levels of NLRP3, ASC, caspase-1 and IL-1β proteins are also elevated (Py et al. 2013). Our *in-silico* and *in-vitro* data support that both FA and PCA favorably bind with the NLRP3 inflammasome pockets and NF-κB protein (Kinra et al. 2021; Doss et al. 2016). Furthermore, the mRNA expression of NLRP3, ASC, Caspase-1, NF-κB and IL-1β, as well as the released IL-1β were significantly reduced in the in-vitro study. Therefore, we decided to explore if the NLRP3 inflammasome pathway is involved in translating the anti-sickness behavior and thereby the antidepressant-like effects of both FA and PCA. Activation of NLRP3, ASC and caspase-1, as well as the assembly of NLRP3-inflammasome are prerequisite to cleavage of IL-1β into its active form in macrophages and microglia. To correlate these changes in the cytokine levels with inhibition of the NLRP3-inflammasome pathway, the protein expression levels of NLRP3, ASC, caspase-1 and IL-1β were quantified. LPS administration significantly upregulated the expression NLRP3, ASC, caspase-1 and IL-1β suggesting the assembly and activation of the NLRP3-inflammasome pathway. These changes in the protein expressions were reduced by the pretreatment of animals with FA in a dose-dependent manner. A similar trend was observed with PCA pretreatment, which significantly downregulated the expression of all four proteins, however, there was no observable dose-dependency, especially for caspase-1 and IL-1β. The effect of all the tested doses of PCA was equally effective against NLRP3 and ASC but in the case of caspase-1 and IL-1β, the median dose and the highest dose were most effective, respectively. Since the activation and assembly of NLRP3-inflammasome are prerequisite to the cleavage of IL-1β into its active form, the downregulation in these proteins directly affected the IL-1β levels in the brain. The levels of IL-1β in the brain homogenates were detected at a significantly high level as compared to the controls and both the pretreatment groups with FA and PCA in a dose-dependent manner, where the higher doses of both the test compounds were most effective in reducing this change.

Although various doses of LPS have been reported to induce sickness behavior, however, the dose selected for this study was based on our earlier published work (Basu Mallik et al. 2016; Mudgal et al. 2019, 2020). Both FA and PCA have well-reported pharmacological properties, however, most of the work on the anti-inflammatory and antidepressant properties is based on low doses and chronic administration (Pei et al. 2016; Mancuso and Santangelo 2014). However, taking the toxicological (LD_50_) and pharmacokinetic (time to C_max_) data into account, we chose a wide dose range (40, 160 and 640 mg/kg, *p.o.*) and an acute administration in this study (Pei et al. 2016; Mancuso and Santangelo 2014). Furthermore, there is a dearth of literature on the activity of these compounds against NLRP3-inflammasome pathway.

The results from OFT, FST and TST were used to assess the changes in behavior post-LPS administration and therefore potential activity of FA and PCA at all three doses. The number of line crossings was used as the parameter to assess the locomotor activity of the animals in OFT. Administration of LPS significantly reduced the number of line crossings which was reversed by the test compounds. The low dose of FA was most effective in increasing the number of line crossings. Among the tested doses of PCA, the highest dose of PCA showed the maximum increase in locomotor activity. Similarly, in FST and TST, LPS significantly increased the immobility period in the animals whereas pre-treatment with test compounds decreased that time at all the tested doses. Although FA did not show any dose-dependent change in the immobility period in FST, the highest dose of PCA was most efficacious which was similar to the locomotor activity changes in the OFT. In TST, although all three tested doses showed an equal response for FA and PCA, the activity of PCA in decreasing the immobility period was better than FA. Fluoxetine was able to reverse the LPS-induced changes in all three behavioral tests although they were significant only in FST and TST. The positive changes in the behavioral parameters indicated that compounds are active against LPS-induced sickness behavior but the underlying inflammatory component still needed to be explored. To assess the biochemical and molecular changes induced by pre-treatment of test compounds, the changes in cytokine production as well protein expression of NLRP3-inflammasome pathway were analysed.

IL-6, a pro-inflammatory cytokine plays an important role in physiology as well as disease state. Administration of LPS is known to elevate the levels of IL-6 in the brain through STAT-3 and GSK-3 signaling (Beurel and Jope 2009). Apart from that, the IL-1β produced in the brain can also induce the synthesis of IL-6 in the microglia via type-1 receptor (IL-1R1) activation and signaling mechanisms such as mitogen-activated protein kinases and NF-κB (Tsakiri et al. 2008). The results obtained from the estimation of IL-6 levels in the brain were evidently in line with these facts. The trend observed in the decrease of IL-6 levels after pre-treatment with test compounds corresponded with that of the decrease of IL-1β levels. All three tested doses of FA and PCA reduced the levels of IL-6 in a dose-dependent manner with a trend similar to that of IL-1β. The highest doses of both the test compounds showed the most amount of reduction in IL-6 cytokine levels.

TNF-α is another major pro-inflammatory cytokine produced in response to TLR stimulation. Recently, it has been shown that P2X7 receptors which are dominant in second signaling for the assembly of NLRP3-inflammasome also induce the release of TNF-α converting enzyme (TACE). TACE converts the TNF-α to its active form (Barberà-Cremades et al. 2017). Therefore, levels of TNF-α in the brain can also be an indirect indicator of NLRP3-inflammasome activation status when correlated with other pro-inflammatory markers. The test compounds at all the tested doses were able to decrease the LPS-induced elevation in TNF- α levels. A dose-dependent reduction in TNF-α was also observed which was very similar to that of IL-1β and IL-6. The higher test doses were able to reduce the levels of TNF-α in a more efficacious manner as compared to the lowest dose of test compounds.

Further, oxidative stress is considered as proximal signals for the activation of NLRP3-inflammasome (Abderrazak et al. 2015). Conversely, the inflammatory environment is known to induce oxidative stress in brain. The production of reactive oxygen species (ROS) in large amounts during inflammatory phase can potentially result in cellular damage. These species can react with cellular membrane polyunsaturated fatty acid to cause lipid peroxidation leading to the production of oxidative products such as malondialdehyde (MDA) (Abdel-Salam et al. 2014). Furthermore, ROS has also been reported to act as a messenger for activation of NLRP3-inflammasome which can be blocked by ROS scavengers and NADPH oxidase inhibitors (Franchi et al. 2012). Both FA and PCA, being polyphenols, have a capability of suppressing the generation of free radicals thereby reducing the rate of oxidative damage. Moreover, these compounds can act as free radical scavengers directly inhibiting oxidative stress-induced damage (Tsao 2010). Reduction of oxidative stress by the test compounds could be a supportive mechanism in alleviating the NLRP3-inflammasome mediated neuroinflammation. Both the test compounds at all three tested doses were able to inhibit the LPS-induced oxidative stress in the mouse brain and all test doses were equally effective against the oxidative stress.

This study highlights the therapeutic potential of FA and PCA in mitigating sickness behavior and associated neuroinflammation induced by LPS in mice. Both FA and PCA effectively alleviated the behavioral symptoms of sickness behavior, enhancing locomotor activity and reducing immobility duration. Importantly, FA and PCA demonstrated anti-inflammatory and neuroprotective properties by reducing proinflammatory cytokines and lipid peroxidation. Recent studies have substantiated the potential of FA and PCA against LPS-induced NLRP3-inflammasome mediated behavioral and molecular changes in mice brain. The results of present study align with recently published reports where FA inhibited inflammasome activation and induced autophagy in THP-1 cells challenged with LPS (Liu et al. 2022). Moreover, PCA also demonstrated potential anti-depressant-like effects via inflammasome inhibition (Lee et al. 2018).

These findings suggest the suitability of FA and PCA as anti-inflammatory and antioxidant agents, particularly relevant in the context of neurodegenerative diseases characterized by underlying neuroinflammation including Alzheimer’s disease, Parkinson’s disease, amyotrophic lateral sclerosis, traumatic brain injury, stroke, multiple sclerosis, systemic lupus erythematous, etc.

In conclusion, this research emphasizes FA and PCA's therapeutic value in countering neuroinflammation and associated pathologies, providing promising avenues for future investigations in the field of neuropsychiatric disorders.

## Data Availability

The datasets generated during the current study are available from the corresponding author upon reasonable request.
